# High eEF1A1 Protein Levels Mark Aggressive Prostate Cancers and the In Vitro Targeting of eEF1A1 Reveals the eEF1A1–actin Complex as a New Potential Target for Therapy

**DOI:** 10.3390/ijms23084143

**Published:** 2022-04-08

**Authors:** Alessandra Bosutti, Barbara Dapas, Gabriele Grassi, Rossana Bussani, Fabrizio Zanconati, Fabiola Giudici, Cristina Bottin, Nicola Pavan, Carlo Trombetta, Bruna Scaggiante

**Affiliations:** 1 Department of Life Sciences, University of Trieste, Via Valerio 28 and Via Weiss 1, 34127 Trieste, Italy; alessandra.bosutti@units.it (A.B.); bdapas@units.it (B.D.); 2 Department of Medical, Surgical and Health Sciences, University of Trieste, Cattinara Hospital, Strada di Fiume, 447, 34149 Trieste, Italy; bussani@units.it (R.B.); fabrizio.zanconati@asugi.sanita.fvg.it (F.Z.); fabiola.giudici@gustaveroussy.fr (F.G.); cbottin@units.it (C.B.); nicpavan@gmail.com (N.P.); trombcar@units.it (C.T.)

**Keywords:** castration-resistant prostate cancer, cytotoxicity, confocal microscopy, eukaryotic elongation factor 1A1, GT75 aptamer, immunohistochemistry, PC-3, PZHPV-7

## Abstract

Although the eukaryotic elongation factor eEF1A1 plays a role in various tumours, there is little information on its prognosis/therapeutic value in prostate carcinoma. In high-grade and castration-resistant prostate carcinoma (CRPC), the identification of novel therapeutic markers/targets remains a priority. The expression of eEF1A1 protein was determined in formalin-fixed, paraffin-embedded prostate cancer and hyperplasia tissue by IHC. The role of eEF1A1 was investigated in a cellular model using a DNA aptamer (GT75) we previously developed. We used the aggressive CRPC cancer PC-3 and non-tumourigenic PZHPV-7 lines. Cytotoxicity was measured by the MTS assay and eEF1A1 protein levels by in-cell Western assays. The mRNA levels of eEF1A1 were measured by qPCR and ddPCR. Higher expression of eEF1A1 was found in Gleason 7–8 compared with 4–6 tissues (Gleason ≥ 7, 87% versus Gleason ≤ 6, 54%; *p* = 0.033). Patients with a high expression of eEF1A1 had a worse clinical outcome. In PC-3, but not in PZHPV-7, GT75 decreased cell viability and increased autophagy and cell detachment. In PC-3 cells, but not in PZHPV-7, GT75 mainly co-localised with the fraction of eEF1A1 bound to actin. Overexpression of the eEF1A1 protein can identify aggressive forms of prostate cancer. The targeting of eEF1A1 by GT75 impaired cell viability in PC-3 cancer cells but not in PZHPV-7 non-tumourigenic cells, indicating a specific role for the protein in cancer survival. The eEF1A1–actin complexes appear to be critical for the viability of PC-3 cancer cells, suggesting that eEF1A1 may be an attractive target for therapeutic strategies in advanced forms of prostate cancer.

## 1. Introduction

Prostate adenocarcinoma (PCa) is the second most common form of solid cancer and the leading cause of cancer-related deaths in men in Western countries [1].

PCa often progresses to severe castration-resistant (androgen-independent) prostate cancer (CRPC) that metastases [2]; metastases are more likely to occur in grade 3 or higher prostate cancer [3].

Treatment options for patients with CRPC remain limited due to resistance to second- and third-line therapies. In addition, current treatments suffer from toxic effects that drastically reduce patients’ quality of life [4].

In mammals, two eukaryotic elongation factor 1A (eEF1A) isoforms exist: the eEF1A1 and eEF1A2 whose genes map on 6q14 and 20q13 chromosomes, respectively [5]. Both isoforms are key players in mRNA translation machinery, carrying the amino-acylated tRNA to the A site in the ribosome. In addition to this “canonical function”, it is known that eEF1As retain several other roles, including cytoskeleton remodeling, chaperone-like/proteosome-mediated protein degradation, and the control of cell growth/death.

In cancer research, eEF1A1 and eEF1A2 proteins have been proposed as possible hallmarks for cell transformation and tumour progression [5]. The eEF1A1 and eEF1A2 proteins have been suggested as possible features of cell transformation and tumour progression. They have also been considered as interesting prognostic factors for some cancers such as breast, lung, colorectal, hepatocellular carcinoma, and leukaemia [5]. We previously observed that eEF1A2 is a potential prognostic factor for prostate adenocarcinomas and plays a role in the development and progression of cancer cells [6]. In addition, eEF1A2 has been shown to be an independent biomarker for prostate cancer risk stratification, because its overexpression is negatively associated with recurrence-free survival [7]. In contrast to eEF1A2, the contribution of eEF1A1 to the phenotypic and molecular changes in human prostate cancer is far less clear [8]. Therefore, in this study, we focused on the determination of eEF1A1 expression in human prostate cancer tissue and investigated the role of eEF1A1 in a poorly differentiated, hormone-independent cell line. To this end, eEF1A1 was targeted by GT75, a 75-nucleotide-long single-stranded DNA aptamer with GT repeats, which we have shown can interact with eEF1A1 and effectively downregulate HCC viability [9]. For several years, we have been focusing on the development and characterization of various GT (guanosine–thymidine) aptameric DNA-based sequences that can inhibit the growth of many human cancer cell lines, including hepatocellular carcinoma cells (HCCs) [9,10,11,12,13,14,15,16].

Our study aimed to investigate eEF1A1 as a biomarker for high-risk prostate cancer and to highlight the possible mechanism of eEF1A1 involvement in tumour progression.

Our data show that the eEF1A1 protein is significantly overexpressed in Gleason ≥7; the highest expression was found in locally undifferentiated cancer cells. In PC-3 cells, used as a model for aggressive CRPC cancer [17], the aptamer GT75 can bind eEF1A1 in vitro, reduce cell viability, and promote cell detachment and autophagy, suggesting that eEF1A1 may be a therapeutic target for advanced prostate cancers. The ability of GT75 to co-localise with the fraction of eEF1A1 bound to actin in the cytoskeleton suggests that the eEF1A1–actin complex plays a role in cancer cell maintenance. Remarkably, no significant effects on cell viability have been observed in the non-tumourigenic PZHPV-7 prostate cells, in which co-localisation of GT75 with the eEF1A1–actin complex is much less evident. Taken together, the reported data indicate the important role of the eEF1A1–actin network in the maintenance of advanced prostate cancer.

## 2. Results

### 2.1. The eEF1A1 Protein Has the Highest Expression in Advanced Prostate Cancer Tissue

**Figure 1:**
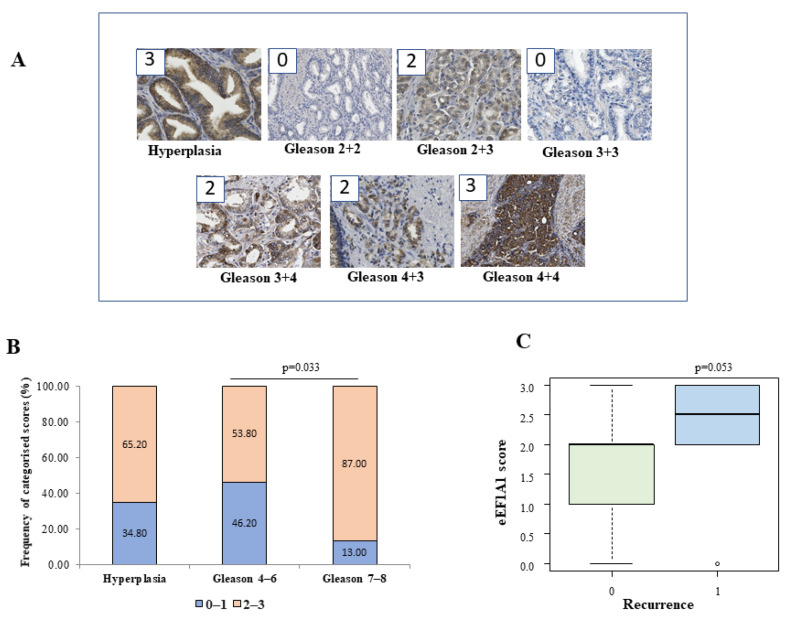
Immunohistochemistry of eEF1A1 expression in tissues. The sample tissues of a cohort of patients were analysed retrospectively, as described in the Materials and Methods. (**A**) Representative images of 20× magnification samples and assigned scores (in boxes). (**B**) Distribution of eEF1A1 protein scores in the groups under study (Hyperplasia *n* = 23; Gleason 4–6, *n* = 26; Gleason 7–8, *n* = 23). (**C**) Boxplot of the distribution of eEF1A1 score with respect to recurrence (1) and no recurrence (0) in cancer patients’ groups (*n* = 44). The *p* values of the Mann–Whitney test for continuous variable or chi-squared test for categorized values are shown.

The characteristics of the patient cohort are shown in Table S1 in the Supplementary Materials. Formalin-fixed, paraffin-embedded tissue samples from 72 patients with prostate cancer and hyperplasia were analysed for protein expression of eEF1A1. We used the previously established IHC method [18] to assess the protein expression of eEF1A1 in prostate cancer tissues with Gleason 4–6 (grade group I, low risk) and Gleason 7–8 (grade group II–IV, intermediate to high risk). Figure 1A shows representative images of eEF1A1 staining and associated scores in different cancer tissue samples and hyperplasia. The eEF1A1 staining was mainly cytoplasmic and in cancer tissues; the strongest intensity (score 3) was found in undifferentiated cancer cells. There were significant differences in the distribution of eEF1A1 scores between the three groups (Figure 1B), both when the expression of eEF1A1 was categorised as 0–1, 2–3 (*p* = 0.04, chi-squared test) and when the variable was considered continuous (*p* = 0.044, Kruskal–Wallis test) (Supplementary Materials Table S2). Pairwise comparisons of eEF1A1 scores were significantly higher in the Gleason 7–8 group than in the Gleason 4–6 group (*p* = 0.03). Interestingly, a positive correlation (simultaneous increase in Gleason score and eEF1A1 values) was found between Gleason and eEF1A1, which was statistically significant (Spearman’s correlation coefficient: r = 0.36; *p* = 0.01). Analysis of eEF1A1 mRNA in 22 tissues of the studied cases showed higher eEF1A1 levels in the Gleason groups compared with the hyperplasia group, although this was not statistically significant (*p* = 0.15) (Supplementary Materials Table S3). Finally, investigation of the possible association between eEF1A1 scores and patient prognosis revealed higher eEF1A1 scores in patients with recurrence (*p* = 0.053, Mann–Whitney test) (Figure 1C and Supplementary Materials Table S4): specifically, 90% of patients with recurrence (9/10) had an eEF1A1 score ≥2 at diagnosis compared with the 61.7% (21/34) of recurrence-free patients.

### 2.2. Targeting of the eEF1A1 Protein by the GT75 DNA Aptamer Reduces the Viability of Undifferentiated Prostate Cancer Cells PC-3

To investigate the role of eEF1A1 in the maintenance of aggressive prostate cancer cells, we used the prostate cancer cell line PC-3 cells, which has been described as androgen-independent with a highly undifferentiated phenotype [17], thus mimicking CRCP cells. The non-tumourigenic human prostate cell line PZHPV-7 served as a control [6]. We have previously observed that the DNA aptamer GT75 in HCC mainly binds to eEF1A1, which is present in the fraction of cytoskeleton/nuclear-enriched protein extract [9]. To demonstrate the same ability in PC-3, we examined the binding of GT75 to eEF1A1 in cell protein extracts using UV cross-linking and a competition assay performed in the presence of the GT27 aptamer, a known eEF1A1 binding competitor [19]. GT75 formed the expected 100 kDa complex and the GT27 competitor efficiently displaced GT75 binding in a dose-dependent manner, even at the lowest molar ratio, indicating the specificity of GT75 to target eEF1A1 (Supplementary Materials Figure S1A). Similar results were obtained for the non-tumourigenic PZHPV-7 cells (data not shown).

**Figure 2:**
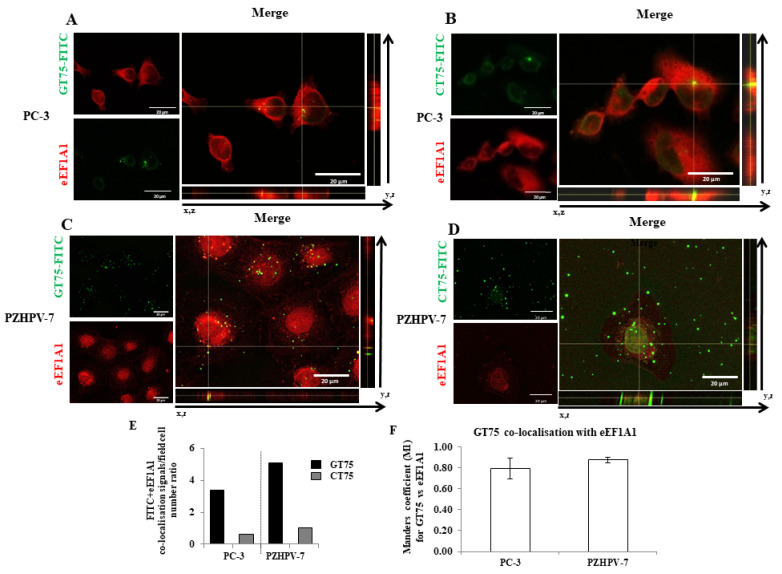
GT75 aptamer co-localisation with eEF1A1 protein in PC-3 and PZHPV-7 cells. Representative images of immunofluorescence confocal microscopy of the GT75-FITC aptamer ((**A**,**C**), green, 150 nM) and control CT75-FITC ((**B**,**D**), green, 150 nM) co-localisation (arrows in orthogonal view) with eEF1A1 protein (red), 3 h after GT75/CT75-FITC transfection in PC-3 and PZHPV-7. (**A**,**B**) Orthogonal views and 3D surface plots show GT75-FITC co-localisation at distinct points with eEF1A1 protein both in PC-3 (**A**) and PZHPV-7 (**C**). (**B**,**D**) Cellular distribution in PC-3 and PZHPV7, respectively, of the control CT75-FITC. Merge of confocal Z-stack images, objective immersion oil, 60×, bars 20 µm. (**E**) Number of co-localisation signals, manually counted in the overlay of different pictures randomly captured in PC-3 and PZHPV-7 transfected by GT75-FITC or CT75-FITC. (**F**) Spatial proximity between GT75-FITC and eEF1A1, in PC-3 and PZHPV-7, estimated by Manders’ co-localisation coefficient M1, are shown as the mean ± SEM. *n* = 9 (PC-3) and *n* = 6 (PZHPV-7). Surface plots are diagrams of three-dimensional data of the indicated cells (arrow) and are shown in grey images.

To investigate the ability of GT75 to also interact with the eEF1A1 protein in cells, cellular co-localisation of an FITC-labelled GT75 (GT75-FITC) was examined by confocal microscopy both in PC-3 cells and in non-tumourigenic PZHPV-7 cells (Figure 2). GT75-FITC/eEF1A1 co-localisation studies were performed with a specific anti-EF1A1 antibody. The aptamer and control were administered at concentrations of 150 nM which, as we have previously found, showed low non-specific toxicity due to the cationic liposome carrier and a specific reduction in cell growth with a strong difference between the two treatments. Orthogonal views of image-captures and 3D surface plots showed that GT75-FITC co-localised with eEF1A1 protein at discrete points within PC-3 (Figure 2A,E). Remarkably, the control CT75-FITC (with CT instead of GT repeats) showed much less discrete point co-localisation (Figure 2B,E). Comparable results were obtained in PZHPV-7 cells (Figure 2C–E). Similar quantitative co-localisation was observed between PC-3 and PZHPV-7 (Figure 2E,F), with the Manders co-localisation coefficient M1 [19] indicating the overlap fraction of the green channel GT75-FITC in the red eEF1A1. Taken together, the above data demonstrate the strict contiguity of GT75 to eEF1A1 in live prostate cancer cells PC-3 and non-tumourigenic PZHPV-7 cells.

**Figure 3:**
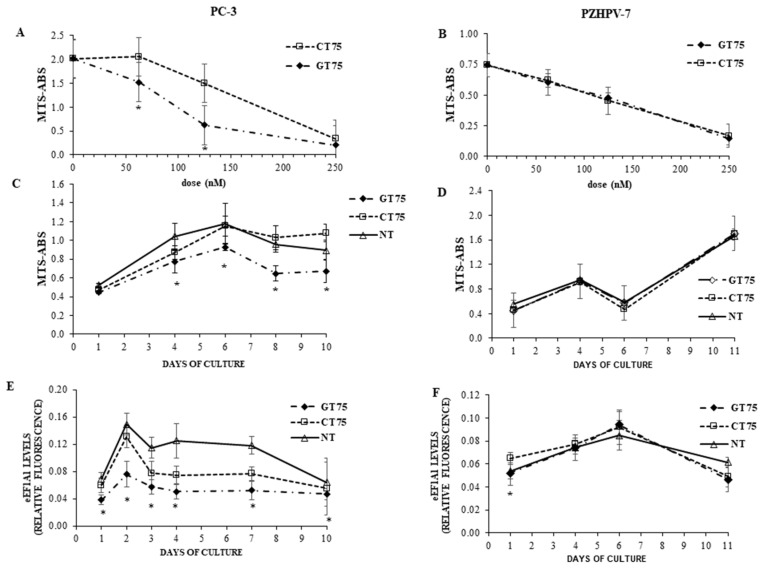
Effects of eEF1A1 targeting by GT75 on cell viability and protein level in PC-3 and PZHPV-7. (**A**,**B**) Dose-dependent effect of the GT75 aptamer and of the control CT75 on cell viability in PC-3 (**A**) and PZHPV-7 (**B**) cell lines evaluated by MTS assay 10 days after aptamer transfection. (**C**,**D**) Time-dependent effect of GT75 (150 nM) and CT75 (150 nM) on cell viability evaluated by MTS assay. (**E**,**F**) eEF1A1 protein levels, in PC-3 and PZHPV-7 cell lines following GT75 (150 nM) or CT75 (150 nM) transfection. eEF1A1 protein levels expressed as fluorescence arbitrary units, have been quantified by in-cell Western assays. * *p* < 0.05 compared with CT75-treated cells; data are shown as the mean ± SD, *n* = 8.

The ability of GT75 to decrease the viability of HCCs, which we had previously observed [9], prompted us to investigate the phenotypic effects of GT75 in PC-3 and PZHPV-7 cells. The viability of PC-3 cells, but not that of control PZHPV-7 cells, was specifically reduced by GT75 treatment in a dose- (Figure 3A,B) and time-dependent (Figure 3C,D) manner compared with CT75 control treatment. In GT75-treated PC-3, eEF1A1 protein levels were reduced compared with CT75-treated cells on all days measured (Figure 3E); in PZHPV-7 cells, the effect was limited to the first day (Figure 3F). In contrast, no decrease at the mRNA level was observed in GT75-treated samples compared with CT75 control (Supplementary Materials Figure S1B).

**Figure 4:**
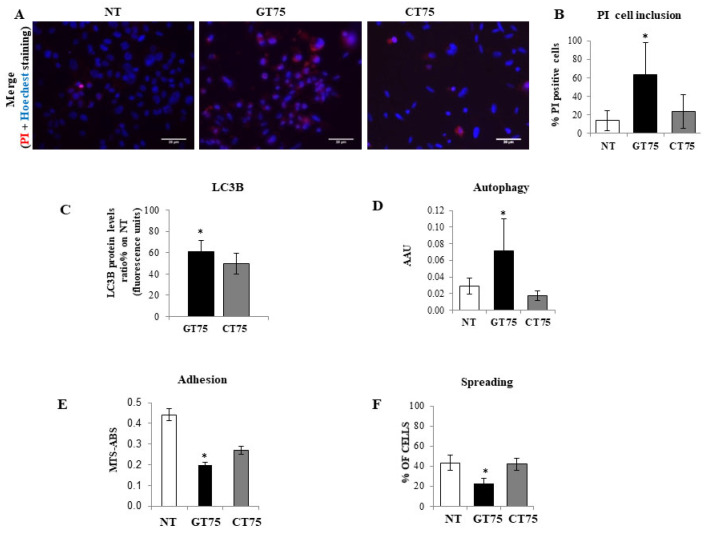
Effects of eEF1A1 targeting by GT75 on cell damage, autophagy, adhesion, and spreading in PC-3 cells. (**A**,**B**) Propidium iodide (PI) nuclei inclusion, indicating cell membrane damage, observed in GT75-treated cells (150 nM) compared with non-treated (NT) and control CT75-treated cells (150 nM) 6 days after transfection. Data in (**B**) are shown as the mean ± SD, *n* = 3. (**C**) Effect of GT75 on the protein level of the autophagy marker LC3B 3 days after transfection. (**D**) Effect of GT75 on the cell autophagy rate evaluated by autophagy colorimetric assay 3 days after transfection. (**E**) Effect of GT75 on cell adhesion compared with NT and control CT75-treated cells 24 h days after transfection. Data in (**C**–**F**) are shown as the mean ± SD, *n* = 8. * *p* < 0.05 compared with CT75-treated cells.

The effect of GT75 on the viability of PC-3 was further investigated by measuring the inclusions of propidium iodide (PI) in the cells. As shown in Figure 4A,B, a significant increase in damaged cells was observed in GT75-treated PC-3 compared with CT75-treated PC-3, as evidenced by the presence of red PI staining in the cytoplasm. In addition, GT75-treated cells showed a higher rate of autophagy compared with CT75-treated controls, as evidenced by both the increase in autophagy marker LC3B (assessed by ICW) and by a colorimetric autophagy assay (Figure 4C,D, respectively), and a significant decrease in adhesion and spreading ability assessed by cell imaging (Figure 4E,F, respectively). A tendency to decrease cell migration was also observed, as assessed by scratch assay (data not shown). Remarkably, no significant effect on apoptosis (Supplementary Materials Figure S2A) was observed in PC-3 cells treated with GT75 compared with CT75, and no major effects were observed in the distribution of cell cycle phases (data not shown).

### 2.3. Targeting of eEF1A1 Protein by the GT75 DNA Aptamer Affects PC-3 Cell Adhesion and Actin/Cytoskeleton Organization

The major non-canonical function of the eEF1A1 protein is to modulate the organisation of the cytoskeleton and the formation of F-actin bundles [20,21,22,23]. Proper actin/cytoskeleton dynamics are critical for cell adhesion and motility. In general, GT75-treated PC-3 cells appeared rounder and vacuolated, with a shrinking morphology and a tendency to detach from the substrate (Supplementary Materials Figure S2B). It is therefore possible that GT75 interacted with actin-bound eEF1A1 in our cellular model and induced structural/functional changes in the cytoskeleton, which, in turn, trigger cell detachment and death.

**Figure 5:**
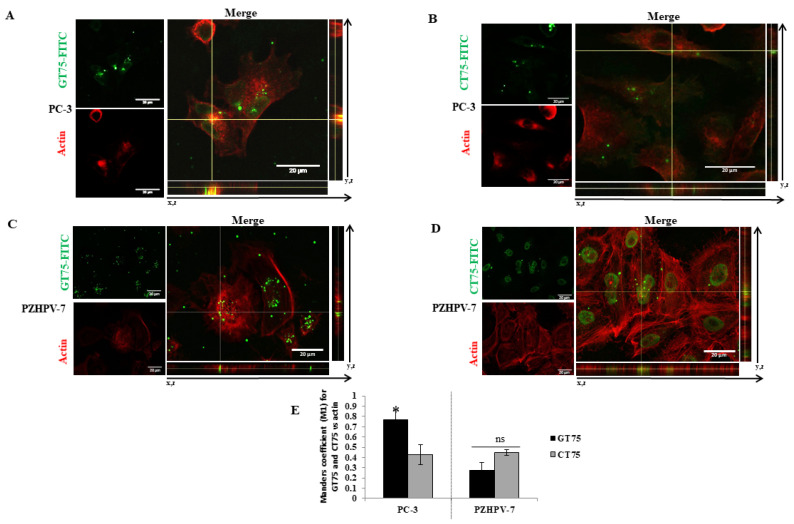
Evaluation of GT75 aptamer co-localisation with actin bundles in PC-3 and PZHPV-7 cells. (**A**–**D**) Representative images of immunofluorescence confocal microscopy of GT75-FITC ((**A**,**C**), green) and CT75-FITC ((**B**,**D**), green) co-localisation (arrows in orthogonal view) with actin protein (red), 3 h after GT75/CT75 (150 nM) transfection in PC-3 and PZHPV-7. Merge of confocal Z-stack images, objective immersion oil, 60×, bars 20 µm. (**E**) Spatial proximity between GT75-FITC and actin quantified by M1 co-localization coefficient of Manders. Data are shown as the mean ± SEM, *n* = 5. * *p* < 0.05 compared with CT75-treated cells; ns, difference statistically not significant.

The above observations and the known contribution of eEF1A1 to modulating cytoskeletal organisation and F-actin bundle formation prompted us to investigate whether GT75 co-localises with the actin fraction bound to eEF1A1. In PC-3, GT75-FITC co-localised with rhodamine–phalloidin-stained actin bundles (Figure 5A). Notably, the M1 co-localisation coefficient of Manders indicates specific co-localisation, because the control aptamer CT75-FITC has significantly lower M1 values (Figure 5B,E). In PZHPV-7 cells (Figure 5C,D), the GT75-FITC/actin co-localisation signal did not appear to be specific, because the M1 value was similar to that measured in CT75-FITC-treated cells (Figure 5D,E). Thus, it appears that GT75-FITC specifically co-localises with the actin-bound eEF1A1 in PC-3, but not in PZHPV-7. Direct binding of GT75-FITC with actin is unlikely, because we have already ruled out this possibility for our GT aptamer series [24]. To confirm GT75-FITC co-localisation with actin-coupled eEF1A1 in PC-3, we examined actin/eEF1A1 co-localisation. eEF1A1/actin co-localisations were found at discrete points in PC-3 cell protrusions, in stress fibres, and around the nuclei (Supplementary Materials Figure S3A). As shown in Supplementary Materials Figure S4A and quantified by the Manders’ M1 co-localisation coefficient (Supplementary Materials Figure S4C), eEF1A1 consistently co-localises with actin in GT75-treated PC-3, This supports the possibility that GT75-FITC interacts with the eEF1A1–actin complex in PC-3. In PZHPV-7, eEF1A1 is also co-localised with actin in untreated (Supplementary Materials Figure S3B), and GT5-treated cells (Supplementary Materials Figure S4B,C). However, the lack of specific GT75-FITC co-localisation with actin (Figure 5) suggests that GT75 interacts mainly with the fraction of eEF1A1 that is not bound to actin.

### 2.4. Depletion of eEF1A1 Protein by siRNA Supported the Role of eEF1A1–actin on Cell Viability in Cancer Cells

**Figure 6:**
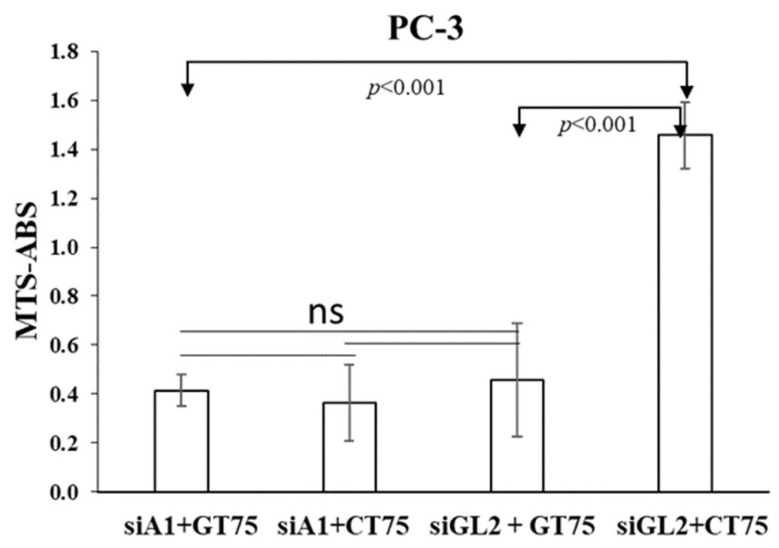
Effects of siRNA and GT75 aptamer co-transfection on PC-3 viability. Evaluation on PC-3 viability of the effects of eEF1A1 targeting by an siRNA (siA1) followed by GT75 administration. Four days after eEF1A1 depletion by siA1 (250 nM), PC-3 cells were transfected by GT75 or the control CT75 (150 nM). Three days after GT75/CT75 transfection, the viability of PC-3 was evaluated by MTS assay; siGL2: cells treated with a control siRNA against luciferase mRNA. Data are shown as the mean ± SD, *n* = 8. The comparisons of PC-3 distributions among the 4 groups were statistically significant (*p* < 0.001, one-way ANOVA test). In particular, the Tukey multiple comparisons of means revealed that the values in the GL2 + CT75 group were significantly higher than both the GL2 + GT75 group (*p* < 0.001) and siA1 + GT75 group (*p* < 0.001). All other pairwise comparisons are not statistically significant.

The above data suggest that GT75 can interact with the eEF1A1–actin complex, possibly causing the phenotypic effects described, which include the decrease in PC-3 viability. To prove the functional role of eEF1A1 in the GT75-induced decrease in PC-3 viability, GT75 was administered to cells after eEF1A1 silencing by siRNA (siA1). Knockdown of eEF1A1 prevented further reductions in cell viability by GT75 (Figure 6, showing a comparison of siA1 + GT75 with siA1 + CT75 and siGl2-GT75), confirming the functional role of eEF1A1 in the GT75-mediated reduction in viability of PC-3. Furthermore, silencing of eEF1A1 *per se* resulted in a specific reduction in viability of PC-3, but not PZHPV-7 cells (Supplementary Materials Figure S5A,B). This suggests that the amount of eEF1A1 in PC-3, but not in PHPV-7, is also important for maintaining cell viability. Remarkably, siA1 significantly reduced the eEF1A1 protein (Supplementary Materials Figure S5C,D) and mRNA levels in both cell lines (Supplementary Materials Figure S6A,B) compared with cells treated with scrambled siRNA (siGL2).

## 3. Discussion

The eEF1A1 protein may play different roles in different tumours. For example, a high expression level of eEF1A1 was found to be a good prognostic factor in patients with colon adenocarcinoma [25], whereas a poor prognosis was found in patients with clear cell renal cell carcinoma [26] and in diffuse large B cell lymphoma [27]. We have found that eEF1A1 protein is overexpressed in advanced prostate cancer tissues, and especially in high-grade cancer cells with respect to low grade tumours (Figure 1). High levels (scores ≥ 2) were frequently found in tissues of high-risk Gleason groups. Overexpression of eEF1A1 was also found in Gleason 4–6 and hyperplasia (54 and 65% of the sample), but it notably increased in Gleason 7–8 (87% of the sample). However, no significant differences in eEF1A1 mRNA expression were found between groups in the respective samples, which is consistent with other findings [25,28]. The fact that the high eEF1A1 levels in tumour tissue and cancer cells are not accompanied by an increase in eEF1A1 mRNA levels may be related to the fact that the eEF1A1 protein has a long half-life; similar observations have been made for the ubiquitin-like protein FAT10 in cancer cells [29].

Specific molecular targeting of tumour proteins that can minimise toxicity to normal cells is a challenge for advanced prostate cancer [30]. In this test, DNA aptamers have been proposed as novel molecular anti-tumour agents. By acting as delivering agents or specific antineoplastic agents in cancer cells, DNA aptamers can inhibit tumour cell growth and proliferation in numerous cancer cell lines [10,31], including prostate cancer cells [6] and cancer stem cells [32]. In addition, some DNA aptamers enter clinical trials [33]. Recently, we have shown that the DNA aptamer GT75, which targets eEF1A1, can inhibit the viability of a panel of human HCCs [9] and the chronic lymphocytic leukaemia cells [34].

In the present study, we investigated the potential of eEF1A1 in sustaining the growth of PC-3 androgen-independent prostate cancer cells representative of CRCP. We have previously shown that eEF1A2 is a potential hallmark for prostate transformation and progression due to its inappropriate expression in prostate cancer cells [11]. We also performed an analysis on eEF1A2 protein levels in the tissues, and found that eEF1A2 was not significantly increased between Gleason 4–6 and Gleason 7–8 groups. Thus, it does not appear to be specifically involved in advanced forms of PCs. In the cell lines used, the presence of both eEF1A isoforms, i.e., eEF1A1 and eEF1A2, cannot completely exclude the binding of GT75 to eEF1A2. However, in PC-3 cells, GT75 resulted in a smaller reduction in eEF1A2 protein level, compared with the eEF1A1 target, which was only visible on day 6 after administration of the aptamer. In addition, no decrease in eEF1A2 level was observed in PZHPV-7 cells at any times (Supplementary Materials Figure S7). Overall, these data suggest that the GT75 mainly targets eEF1A1. Here, we expand our knowledge of the role of eEF1A1 in PC-3 cells, because its contribution is far less clear than that of eEF1A2. Our data showed that the GT75 aptamer can co-localise with (Figure 2) and bind to eEF1A1 (Supplementary Materials Figure S1A). This, in turn, causes PC-3 to reduce cell viability, promote cell death, activate cell autophagy, and impair cell adhesion and cell spreading (Figure 3). Notably, most of these functions attain the eEF1A bound to the cytoskeletal/nuclear compartment [35], whereas the soluble cytoplasmic fraction is mainly involved in mRNA translation (canonical function). In agreement with data obtained in HCC lines [31], there was no significant effect on cell cycle phases (data not shown). Regarding apoptosis, we observed a slight upregulation of apoptosis in HCCs, which was not evident in PC-3 (Supplementary Materials Figure S2A), probably reflecting cell-specific phenotypic differences.

Consistent with the observation in tissues (hyperplasia versus Gleason 7–8), high levels of eEF1A1 protein were also found in non-tumourigenic PZHPV-7 cells. Indeed, whereas GT75 specifically reduces the viability of PC-3 cells, this is not the case in the non-tumourigenic PZHPV-7 cell line (Figure 2B,D). Our data may provide some possible explanations for this observation: eEF1A1 co-localises with GT75 in both PC-3 and PZHPV-7 (Figure 2); however, in PC-3 cells, GT75 is co-localised with actin, whereas in PZHPV-7 cells, GT75 did not appear to specifically co-localise with actin (Figure 5); both in PC-3 and PZHPV-7, eEF1A1 co-localized with actin, but in PC-3, this appears more evidently with a distribution at the cell periphery (see Figures S3 and S4). Therefore, it is reasonable to deduce that the eEF1A1–actin complex is specifically targeted by GT75 in PC-3 but not in PZHPV-7. Actin is important for many cellular processes such as cell adhesion, spreading [36] and autophagy [37]; therefore, the co-localisation of eEF1A1 with actin could be particularly important and influence cell adhesion/spreading and trigger autophagy, as we observed in PC-3 (Figure 4). The fact that no effect of GT75 on the eEF1A1 level and cell growth was shown in PZHPV-7 raises the possibility that in PC-3, other factors may increase the affinity of eEF1A1 for GT75, as recently shown in neuroblastoma for eEF1A1 and SMAD4 [27].

The fact that in PC-3, the siRNA-mediated silencing of eEF1A1 prevents further effects of GT75 on cell number reduction, suggests the functional role of eEF1A1 in cell viability (Figure 6). Furthermore, the observation that the silencing of eEF1A1 *per se* reduces the viability of PC-3 (Supplementary Materials Figure S4) suggests that, overall, eEF1A1 protein levels are also important for cell viability. In PZHPV-7, despite a significant reduction in the eEF1A1 protein level after silencing, no specific effects on cell viability were observed (Supplementary Materials Figure S4). This suggests that eEF1A1 plays a different functional role in PZHPV-7, at least in terms of its non-canonical functions.

In this study, we have showed, for the first time, that actin-bound eEF1A1 maintains the viability of the aggressive prostate cancer cells. Overall, our data suggest that the differential phenotypic effects of GT75 in PC-3 compared with PZHPV-7 are due to: (1) the preferential co-localisation of GT75 with the eEF1A1–actin complex in PC-3 but not in PZHPV-7; and (2) the differential dependence of cell viability on eEF1A1 levels. The fact that eEF1A1 appears to be essential for the survival of the tumour cell line PC-3, but not for the non-tumourigenic PZHPV-7 cell line, raises the possibility that the targeting of eEF1A1 preferentially affects prostate tumour cells compared with non-tumourigenic cells, which could overcome the problem of significant side effects of available treatments for CRCP [4]. The negligible specific effects of GT75 in the non-tumorigenic PZHPV-7 cells are consistent with the lack of a major effect of the GT aptamers we previously developed for the viability of other non-tumourigenic cells [10,12,14,24]. Confocal microscopy data show that in PC-3, the cytoplasmic fraction of eEF1A1 involved in protein elongation is not bound by the GT75 aptamer (Figure 2). Indeed, GT75/eEF1A1 co-localisation is restricted to discrete regions and not scattered throughout the cytoplasm. This suggests an interaction of GT75 with the fraction of eEF1A1 localised in the cytoskeletal/nuclear compartment, which is consistent with our previous studies using the analogous aptamer GT27 in leukaemic cells [24]. Our data show co-localisation of the cytoskeletal/nuclear fraction of eEF1A1 with actin which, in turn, is associated with autophagy [37]; thus, it is plausible that the targeting of this eEF1A1 fraction may influence autophagy, as shown by our data (Figure 4). A possible role of eEF1A1 in maintaining pro-metastatic potential has been suggested. Recently, a highly conserved lncRNA called MALAT1 was found to promote pro-metastatic potential in breast cancer cells by upregulating EEF1A1 expression via epigenetic modulation of the promoter [38]. The novelty of this work, however, is that in prostate cancer, this appears to be due to the eEF1A1–actin complex. In PC-3, GT75 appears to affect the functions of eEF1A1 mainly through its binding. Indeed, no dramatic effects on eEF1A1 expression are evident, because GT75 does not reduce eEF1A1 mRNA levels (data not shown) and moderately, although significantly, reduces the protein level (Figure 3E) for all time points tested. The lack of effects at the mRNA level is consistent with our previous data obtained in a panel of human HCCs [9]. However, although eEF1A1 protein levels were not significantly decreased in HCCs [9], we observed a statistically significant difference here. This apparent contradiction could simply be because we used the semi-quantitative Western blotting technique in HCCs, whereas here we used the more sensible and quantitative ICW assay. Interestingly, despite this methodological difference, the decrease in eEF1A1 protein level we observed here was 25% (average decrease over all time points considered (Figure 2E), which is not so far from the 20% we observed in HCCs [9,39]. In PZHPV-7, the decrease in eEF1A1 was also present but limited to the first day (Figure 3F). As noted above, this observation suggests the possibility of a lesser dependence of PZHPV-7 on a decrease in eEF1A1 levels for cell viability, as demonstrated by siRNA-mediated silencing effects (Supplementary Materials Figure S4A,B). *EEF1A1* mutation was detected in HCC tumour samples [40]. We thus cannot rule out the possibility that in PC-3 cells, but not in PZHPV-7, the EEF1A1 gene is mutated or eEF1A1 protein is post-translationally modified, which can confer to the protein more affinity to complex with actin to promote cell survival and invasion.

## 4. Materials and Methods

### 4.1. Human Prostate Tissues and Immunohistochemistry

Human prostate tissue was retrospectively selected by pathologists. The tissues came from patients who had signed a consent form to donate their tissue for research purposes. The human tissue study was approved by the University of Trieste Ethics Committee No. 101 on 12 April 2019. A total of 26 cases of Gleason 4–6, 23 cases of Gleason 7–8, and 23 cases of hyperplasia were collected from the clinical data of patients admitted at the Hospital of Cattinara for prostate resection for cancer or TURP from 2012 to 2014, and were studied retrospectively. Patients were male (100%) with a mean age ± SD in the high-risk group: 66 ± 5.5 Gleason (3 + 4), 68 ± 4.1 Gleason (4 + 3), and 70 ± 3.6 Gleason (4 + 4). Patients with a previous cancer diagnosis were excluded. Tissue was processed at surgical resection for diagnostic purposes and patients had not previously started antiandrogenic therapy. The characteristics of the cohort studied are detailed in Table S1 of Supplementary Materials. The histopathological diagnosis was confirmed by the expert pathologist who selected tissues for eEF1A1 IHC or the mRNA extraction. Immunohistochemical analysis and tissue scores were evaluated as previously described [11,18]. Immunohistochemical staining of A1 protein was performed in consecutive paraffin-embedded sections of prostate tissue. Briefly, the score was assigned according to a semiquantitative analysis considering the average intensity of the signal coming from all the stacks of a single image. Images were acquired with Leica DM 2500 20× magnification lens. Staining intensity was graded from 0 to 3 (0 = no expression, 1 = weak, 2 = moderate, and 3 = strong). The extent was graded from 1 to 3 (1 = 1–30%, 2 = 31%–60%, 3 = 61%–100% of cells). The extent scores were added to obtain a mean composite score. Patients with composite scores of 0–1 were classified into the low-eEF1A1-expression group, and those with scores of 2–3 were included in the high-eEF1A1-expression group. The results were analysed by an expert pathologist who was blinded to the clinical data.

### 4.2. Cell Cultures

Non-tumourigenic PZHPV-7 cells were cultured in KSFM medium supplemented with specific EGF growth factor and newborn calf serum, as recommended by the manufacturer (Invitrogene, Thermo Fisher Scientific, Waltham, MA, USA). The highly aggressive, non-androgen-responsive cells of the prostate adenocarcinoma phenotype PC-3 were cultured in RPMI-1640 medium supplemented with 2 mM L-glutamine, 10 U/mL penicillin, and 10 µg/mL streptomycin (Euroclone, Milan, Italy) and 10% foetal bovine serum (Euroclone, Milan, Italy). The cells grew at 37 °C in a humidified atmosphere with 5% CO_2_. The cells were used for the experimental set-up when they had reached about 70% confluence. The cell lines from ATCC were a generous gift from Prof. G. Manfioletti, University of Trieste.

### 4.3. Aptamer Transfections

PC-3 cells (9 × 10^3^ cells/100 µL, 100 µL per well) and PZHPV-7 cells (11 × 10^3^ cells/100 µL, 100 µL per well) were seeded in complete RPMI-1640 medium and KSFM medium, respectively, in a 96-well microplate. At 24 h post-seeding, the medium was removed and the GT75 aptamer or CT75 control (Eurofins MWG Operon, Genomics, Ebersberg, Germany) was added to Optimem media (Invitrogene, Thermo Fisher Scientific, Waltham, MA, USA) using Lipofectamine 3000 (Invitrogene, Thermo Fisher Scientific, Waltham, MA, USA), in accordance with the manufacturer’s recommendations and using a weight/volume ratio of oligomer/transfection agent of 1.15:1. The concentration range of GT75 or CT75 (62.5–250 nM) was chosen following one of our previous studies. The sequences of the GT75 aptamer and its CT75 control were as previously reported [9]. Twenty-four hours after transfection, the Optimem medium was removed and replaced with a complete RPMI-1640 medium or KSFM medium. The cells were observed for several days. Six days after transfection, the complete medium was replaced with a fresh medium. To test the effects of GT75 and CT75 on EEF1A1 mRNA levels, PC-3 cells (2.4 × 10^5^ cells/2.5 mL, 2.5 mL per well) and PZHPV-7 cells (3 × 10^5^ cells/2.5 mL, 2.5 mL per well) were seeded in 6-well plates and the effects of transfection were assessed in the 1st, 3rd, and 6th wells.

### 4.4. SiRNA Transfections

For silencing eEF1A1, we used an siRNA (siA1) and a control siRNA (siGL2) directed against the luciferase mRNA we had previously selected [11]. PC-3 cells (9 × 10^3^ cells/100 µL, 100 µL per well) and PZHPV-7 cells (11 × 10^3^ cells/100 µL, 100 µL per well) were seeded in 96-well plates containing complete RPMI-1640 media and KSFM media, respectively. After 24 h, siRNA transfection (250 nM, Eurofins MWG Operon, Genomics, Ebersberg, Germany) was performed as described for aptamers. To test the effects of siA1 on EEF1A1 mRNA levels in PC-3 and PZHPV-7, the same conditions as described for aptamers were used.

### 4.5. Real Time RT-PCR and Digital Droplet RT-PCR

At selected endpoints after aptamer and/or siRNA transfections, total RNA was extracted (Sigma Chem. Co., Darmstadt, Germany) and 1 µg of total RNA was reverse-transcribed in the presence of random primers and MuLV reverse transcriptase (Thermo Fisher Scientific, Waltham, MA, USApplera). *EEF1A1* mRNA levels were then determined by real-time PCR (Applied Biosystems, Foster City, CA, USA), as previously described [9]: 28S rRNA was used as the housekeeping gene. The absolute quantification of eEF1A1 mRNA levels was determined from tissue samples using the QX200 Droplet Digital PCR System (ddPCR), because ddPCR has higher precision and sensitivity. Total RNA was extracted for each sample from 10 slices of cancer tissue, 6 mm in diameter and 10 μm thick, using the RNeasy FFPE kit (Qiagen GmbH, Hilden, Düsseldorf, Germany) after standard deparaffinisation with xylene-mediated dewashing. Reverse transcription was performed using the iScriptTM gDNA Clear cDNA Synthesis Kit (Bio-Rad, Milan, Italy) to avoid contamination with genomic DNA, and cDNA amplification was performed using expression primers that performed a probe assay for eEF1A1 FAM (200 nM) (Bio-Rad) and GAPDH HEX (200 nM) (Bio-Rad, Milan, Italy). Subsequently, the fluorescence of the plate containing the samples was measured in red in the QX200™ Droplet Reader instrument. The data were normalised to the GAPDH mRNA content and analysed using QuantaSoft™ software (Bio-Rad).

### 4.6. Cell Viability, Autophagy, and Cell Damage

Cell viability was assessed by the MTS assay using the Cell Titer 96 Aqueous One Solution Assay (Promega, Madison, WI, USA) at an absorbance of 490 nm, according to the manufacturer’s recommendations at different time points after aptamer transfection and using different aptamer concentrations (62.5–250 nM). Similar conditions were used to test the effects of siRNA (250 nM) on cell viability. For combined aptamer/siRNA transfection, four days after eEF1A1 depletion by siRNA against eEF1A1 (siA1, 250 nM) PC-3 cells were co-transfected with GT75 (150 nM) or CT75 (150 nM). Three days after GT75/CT75 transfection, the viability of PC-3 was assessed using the MTS assay.

Autophagy was assessed by consecutive cell staining performed with neutral red (NR), and crystal violet (CV) (Sigma Aldrich, Darmstadt, Germany), and by an independent MTS assay (Sigma Aldrich, Darmstadt, Germany) [39].

Briefly, PC-3 cells (10 × 10^4^ cells) were first stained with NR (30 μg/mL) at 37 °C for 2 h and then washed twice with PBS 1×. The cells were then fixed and eluted NR with an alcoholic 1.0% *v*/*v* acetic acid solution. After NR reading (540 nM), a solution of crystal violet (0.02% *w*/*v*) was added; cells were then incubated for 5 min at room temperature. After incubation, cells were washed twice with distilled water, and before CV reading (585 nM), the CV staining was eluted by adding a 50% *v*/*v* ethanol 0.1 M sodium citrate solution. Cell survival was determined independently using the MTS assay, as previously described. Results are expressed as arbitrary autophagy units (AAUs) calculated using the following formula: NR (OD values)/[MTS (OD values) + CV (OD values)/2], where NR represents cell survival as determined by neutral red, MTS represents cell survival as determined by the amount of formazan product (MTS assay), and CV represents cell survival as determined by crystal violet. All measurements were performed spectrophotometrically using the Synergy H1 HYBRID Fluorimeter Plate Reader (BioTek, Winooski, VT, USA) at the optimal time point of day 3 after aptamer transfection (150 nM).

Cell damage was assessed by the uptake of propidium iodide (10 µg/mL) and counterstaining of cell nuclei with Hoechst 33,258 (Sigma-Aldrich, Germany). In parallel, an “In-Cell Western” assay was performed in another plate to determine the changes in the autophagy marker LC3B protein (Invitrogene, MA5-37852) in response to aptamer treatments (150 nM) in PC-3 cells (0.9–1 × 10^4^ cells), according to the manufacturer’s instructions. Day 6 and 3 after aptamer transfection (150 nM) were the optimal time points for PI staining and LC3B quantification, respectively.

### 4.7. Cell Adhesion/Spreading Assays

Cell adhesion/dispersion experiment: PC-3 cells (6.4 × 10^4^ cells) were transfected in suspension with GT75 or CT75 (150 nM). Forty-five minutes later, the cells were plated out and allowed to grow for 24 h (optimal time to see the effect). The number of attached cells was then determined using the MTS assay (CellTiter 96 Aqueous One Solution Assay, Promega, Madison, WI, USA). Cells were then fixed with cold ethanol for 5 min at room temperature, dried, stained with 1% methylene blue in water, and photographed. Spreading cells were identified by their flat and elongated shape, in contrast to non-spreading cells, which were roundish. Cells were observed under an inverted microscope and counted in five random field exposures. The number of cells was expressed as a percentage of the total cells.

### 4.8. UV-Crosslinking Assay

The cell total nuclear extract preparation and UV-crosslinking assay procedure were conducted as previously described [14,16]. Briefly, 3 μg of total nuclear extract and GT75 (2 ng) labelled with (^32^P) ATP aptamer were incubated for 25 min at room temperature in the presence of a molar excess of poly (dI-dC) and CT27 (non-specific competitor), together with different fold molar excess of unlabelled GT27 (specific competitor). After incubation at room temperature for 1 h, the samples were irradiated at 302 nm for 10 min with a UV trans-illuminator (BioRad Laboratory, Milan, Italy), and the UV-crosslinking products were analysed on a 10% SDS polyacrylamide gel.

### 4.9. Confocal Immunofluorescence Microscopy Analysis

To demonstrate internalisation of the aptamer into the target cells, cells PC-3 (5.8 × 10^4^ cells) and PZHPV-7 (7.5 × 10^4^ cells) were seeded in their complete medium on a glass coverslip in a 24-well microplate. The next day, cells were transfected with GT75 (150 nM) conjugated to fluorescein (FITC) or negative control CT75 (150 nM), and conjugated to FITC, as described above. The cells were then fixed and examined for confocal microscopy with immunofluorescence. Three hours after transfection was the optimal time for successful visualisation of the aptamers in the cell; at longer time points, the FITC signal was no longer detectable.

To detect eEF1A1 and/or actin, PC-3 (5.8 × 10^4^ cells) and/or PZHPV-7 (7.5 × 10^4^ cells) cells were placed on a glass coverslip in a 24-well plate and subjected to transfection (150 nM) with GT75 or CT75 (with or without FITC labelling), as described above. Three hours after transfection (in analogy to the aptamer uptake studies), the medium was removed, and the cells were fixed with 4% paraformaldehyde (*w*/*v* in PBS) and permeabilised with 0.1% Triton X-100 (*v*/*v* in PBS). Cells were incubated in blocking solution (1% BSA (*w*/*v* in PBS)) for 1 h before incubation (1 h at room temperature) with primary antibodies against eEF1A1 protein (mouse monoclonal antibody EF-Tu, code: sc-21758, Santa Cruz Biotechnologies, Santa Cruz, CA, USA). Alexa Fluor 488 goat anti-rabbit IgG (Molecular Probes/Invitrogen) was the secondary antibody. Rhodamine phalloidin staining (Molecular Probes/Invitrogen) was used to visualize filamentous and globular actin. The nuclei were co-stained with Hoechst 33,258 solution (Sigma, Darmstadt, Germany). After immunostaining, coverslips were mounted on microscope slides with fluorsave (Invitrogene) mounting media. Confocal microscopic images were taken using a Nikon C1si confocal microscope equipped with an argon laser (457, 477, 488 and 514 nm lines), a 561 nM, and a 640 nM diode laser, and a 60×/1.4 (NA) plan apochromat oil immersion objective. Series of optical images were acquired with a resolution of 0.15 μM by sequential scanning of the lines to avoid potential crosstalk phenomena between fluorophores. Images were acquired while maintaining the same acquisition settings for laser intensity, pmt gain, pinhole aperture (33 μM) and pixel dwell time to obtain comparable signals between control and treated conditions. The autofluorescence signal was tested with the inclusion of a negative control antibody stain (IgG). Images were processed for z-projection at maximum intensity, and image processing was then performed using Fiji win32 (ImageJ). The co-localisation coefficient (Manders’ Coefficient M1) was determined using Coloc2 (Fiji’s plugin for co-localisation analysis), with the threshold set manually. In each experiment, at least 12 cells per field were analysed. Experiments were performed in triplicate. The regions of interest (ROIs) were chosen so that only the cell areas were selected.

### 4.10. In-Cell Western (ICW)

ICW experiments were performed in 96-well plates after transfecting either aptamer (150 nM) or siRNA (250 nM). At the end of each experimental analysis, the medium was drained, and the cells were fixed in 3.7% formaldehyde in phosphate-buffered saline (PBS) 1× and incubated for 10 min at room temperature. The fixative was removed, and the cells were washed three times with TBS 1×. After washing, the cells were incubated with 0.1% Triton^®^ X-100 in PBS for 10 min at room temperature and washed three times with TBS 1×. After blocking with 1% BSA (*w*/*v* in PBS) for 1 h, the cells were incubated with primary antibodies at a dilution of 1:200 in 1% BSA (*w*/*v* in PBS) against eEF1A1 or at a dilution of 1:1000 in 1% goat serum against LC3B proteins (mouse monoclonal antibody: Invitrogene, Life technologies MA5-37852) for 1 h at room temperature. After washing three times with TBS 1×, incubation with secondary antibody (Molecular Probes/Invitrogen) Alexa Fluor 568 goat anti-mouse IgG was performed for 1 h at room temperature. After 1 h incubation, cells were washed three times with TBS 1×. Immunofluorescence measurements were performed in 100 µL PBS solution using the appropriate filters. After the first measurement, cells were counterstained with a filtered solution of Hoechst 33,258 (0.5 µL/mL in H_2_O) (Sigma Aldrich, Darmstadt, Germany) for 30 min at room temperature. Hoechst 33,258 was then removed, the cells were washed, and the measurement was performed in 100 µL PBS 1× solution, using the appropriate filters. The maximum excitation and emission peaks were determined by evaluating the full spectra for each antibody-conjugated fluorochrome. Fluorescence channels 578/610 nm were used for eEF1A1/LC3B detection and 345/478 nM (Synergy H1 HYBRID Fluorimeter Reader, BioTek, Winooski, VT, USA) for the quantification of Hoechst nucleic acid staining. The quantification of the Hoechst nuclei staining served as a normalisation for the determination of the eEF1A1 or LC3B protein levels in each sample.

### 4.11. Apoptosis

PC-3 (3 *×* 10^5^ cells) cells were seeded in 6-well plates and aptamer transfection was performed as described in the Materials and Methods. At specific time point (6 days after transfection), cells were harvested for apoptosis analysis. The cells were incubated in annexin-V/FITC and propidium iodide labelling solution (Bender Medsystem) for 30 min, washed, and then resuspended in 0.5 mL of PBS 1×/BSA (bovine serum albumin) 0.5% and analysed by FC500 (Beckman Coulter, Venezia, Italy) flow cytofluorimeter. The experiments were performed in triplicates.

### 4.12. Statistical Analysis

All statistical analyses on tissue samples were carried out in R software for statistical computing (RStudio: Integrated Development for R. RStudio, Inc., Boston, MA, USA, URL http://www.rstudio.com/ (accessed on 27 February 2022)). Statistical significance was set at *p* < 0.05. The Shapiro–Wilk test was applied to continuous variables to check for distribution normality. Quantitative variables were reported as the median with range (min–max) or mean ± standard deviation, depending on the distribution. Categorical variables are reported as absolute frequencies and/or percentages. Continuous variables were compared by Mann–Whitney test (and Kruskal–Wallis test) or Student’s *t*-test (and ANOVA), depending on data distribution and the number of groups. Bonferroni post hoc analysis for more than two groups was performed. For the assessment of correlation between eEF1A1 levels and Gleason scores, Spearman’s correlation coefficient was calculated.

All data on cell cultures were expressed as the mean ± S.D or SEM of replicates or independent experiments. Statistical significance was evaluated by the Student’s *t*-test.

## 5. Conclusions

Further studies are needed to show that eEF1A1 may be an independent predictor of the pathological stage, metastatic disease, biochemical recurrence, and cancer-specific survival in prostate cancer. Despite the small number of patients, this study strongly suggests that eEF1A1 overexpression plays a role as a marker of prostate cancer aggressiveness as evidenced by recurrence and high score. Finally, the data overall point to a relevant role of the eEF1A1–actin complex in sustaining the viability of aggressive prostate cancer cells. These observations open new perspectives for further studies aimed at developing new therapies targeting eEF1A1.

## Data Availability

The data that support the findings of this study are available from the corresponding author upon reasonable request.

## Supplementary Materials

The following are available online at https://www.mdpi.com/article/10.3390/ijms23084143/s1.
