# Supplementary material for: High eEF1A1 Protein Levels Mark Aggressive Prostate Cancers and the In Vitro Targeting of eEF1A1 Reveals the eEF1A1–actin Complex as a New Potential Target for Therapy
Source: Int J Mol Sci. 2022 Apr 8;23(8):4143. doi: 10.3390/ijms23084143 (PMC9027132; doi:10.3390/ijms23084143)
Supplement: Supplementary file 1 [file ijms-23-04143-s001.zip › Figure S1.pdf]

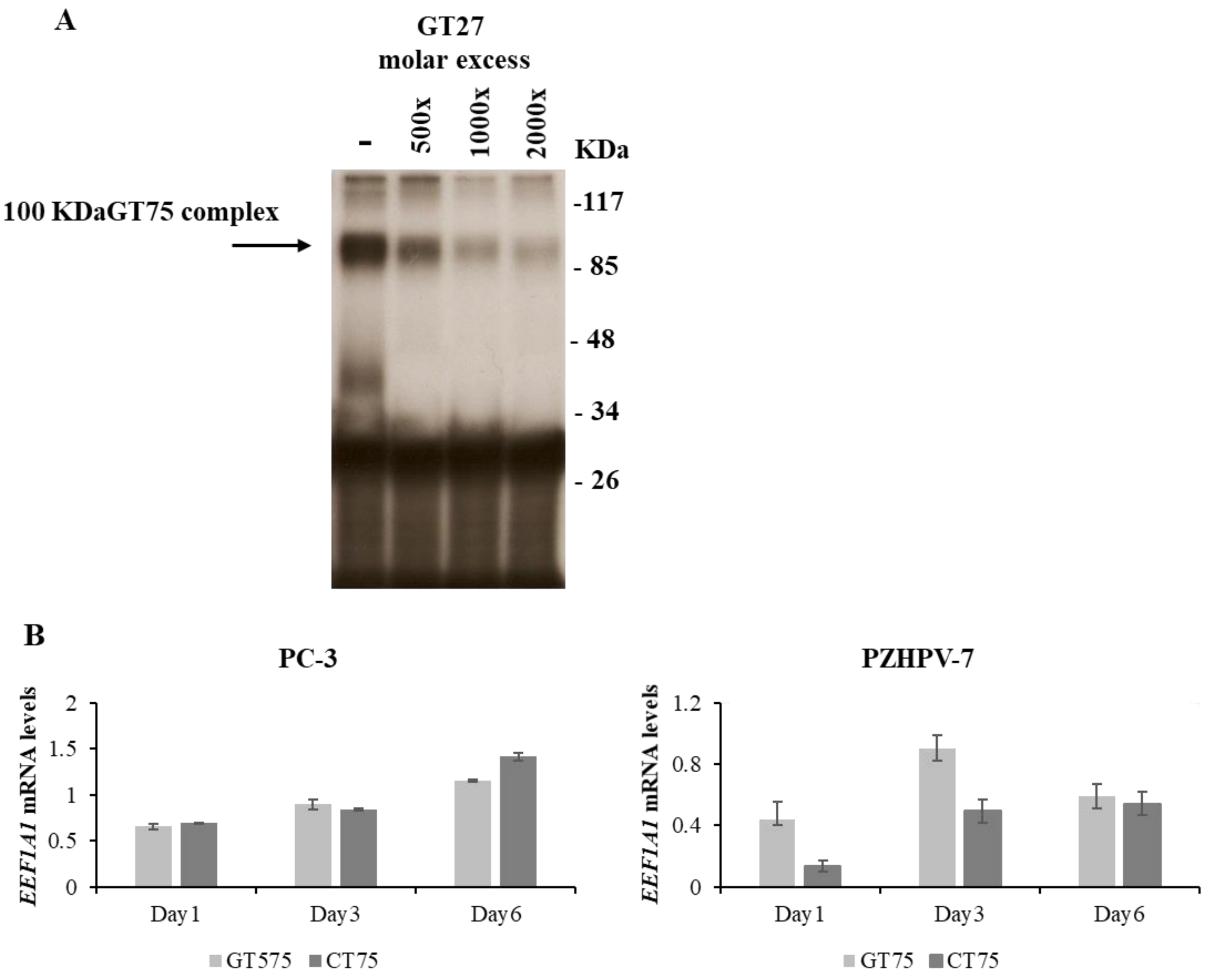

Figure S1: A: GT75 aptamer forms a complex of about 100 kDa in prostate cancer cells. GT75 was efficiently displaced by a molar excess of the aptamer GT27, thus confirming the specific binding to eEF1A1. B: In PC-3 and PZHPV-7 cells, the GT75 aptamer and the CT75 control were administered to cells (150nM). The cells were harvested for the RNA extraction after one, two, and three days. Analysis was performed by real time RT-PCR as described in Materials and methods. Data are shown as mean ±SD of three replicates
