# Supplementary material for: High eEF1A1 Protein Levels Mark Aggressive Prostate Cancers and the In Vitro Targeting of eEF1A1 Reveals the eEF1A1–actin Complex as a New Potential Target for Therapy
Source: Int J Mol Sci. 2022 Apr 8;23(8):4143. doi: 10.3390/ijms23084143 (PMC9027132; doi:10.3390/ijms23084143)
Supplement: Supplementary file 1 [file ijms-23-04143-s001.zip › Figure S2.pdf]

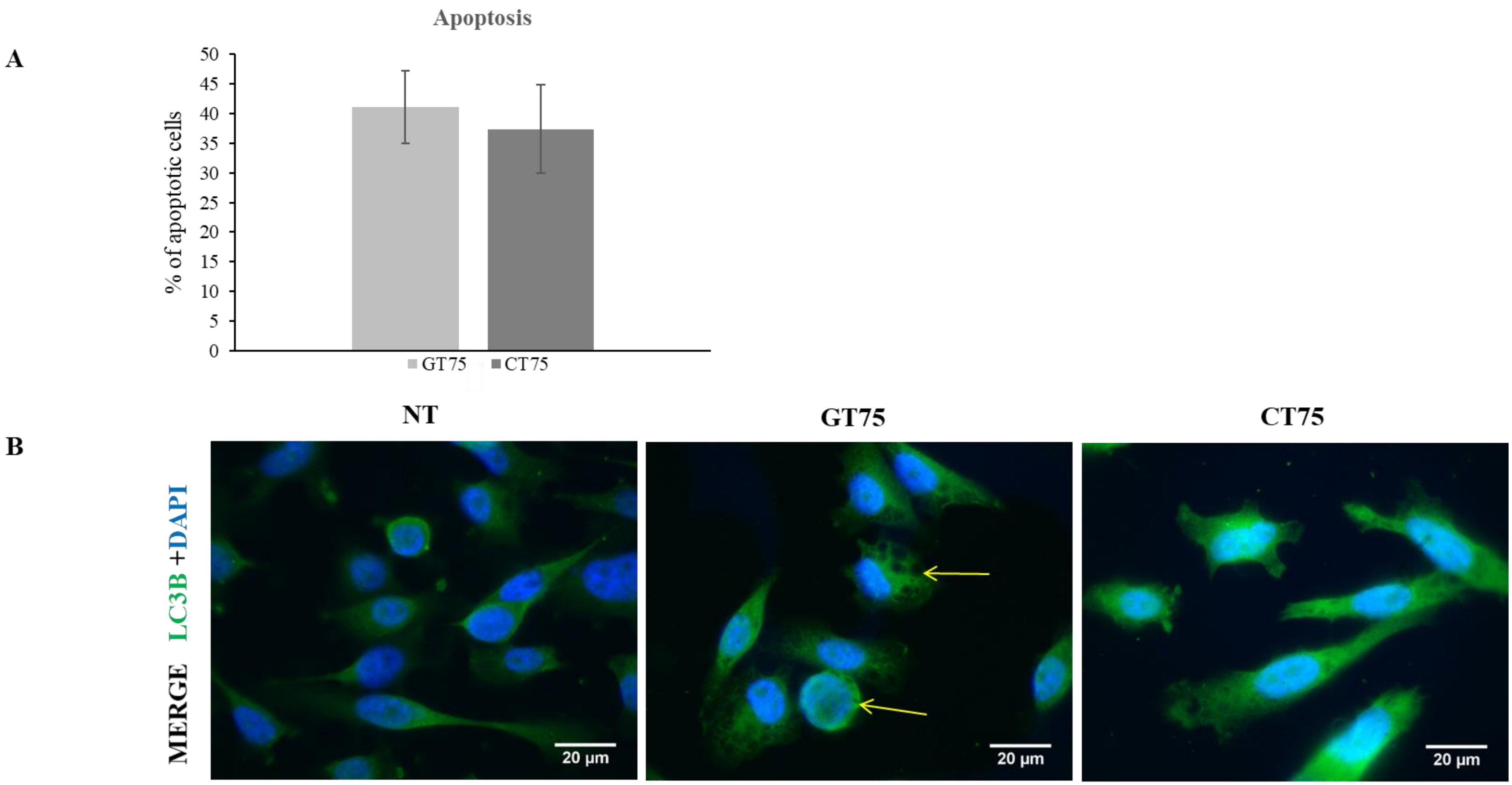

Figure S2: A: Effect of GT75 aptamer and of CT75 control (150 nM) on cell apoptosis in PC-3 cells six days from transfection, evaluated by annexin V. B: GT75-treated (150 nM) PC-3 cell morphology six days from aptamer transfection. Cells appeared more rounded and vacuolated (arrows), and with a shrinking morphology. NT, not treated cells; GT75, transfected cells with aptamer GT75; CT75, transfected cells with control CT75. Representative images of cell morphology, (LC3B, green+DAPI, blue), objective immersion oil, 100x.
