# Supplementary material for: High eEF1A1 Protein Levels Mark Aggressive Prostate Cancers and the In Vitro Targeting of eEF1A1 Reveals the eEF1A1–actin Complex as a New Potential Target for Therapy
Source: Int J Mol Sci. 2022 Apr 8;23(8):4143. doi: 10.3390/ijms23084143 (PMC9027132; doi:10.3390/ijms23084143)
Supplement: Supplementary file 1 [file ijms-23-04143-s001.zip › Figure S3.pdf]

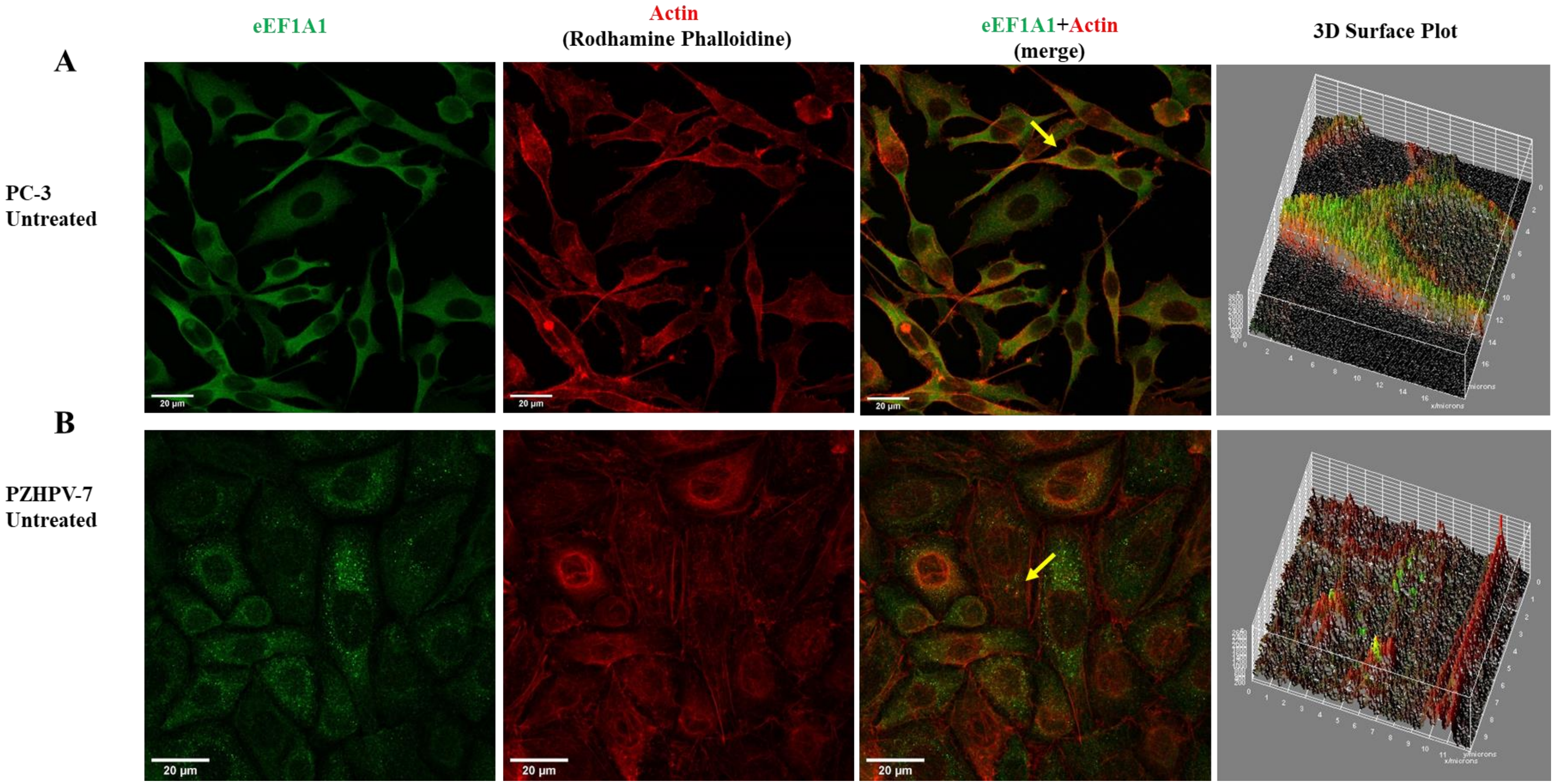

Figure S3: A: Representative immunofluorescence confocal images showing the co-localisation of eEF1A1 (green) with actin (red) in untreated PC-3; confocal Z-stack images, objective immersion oil, 60 x, bars 20µm. B: As in A but with PZHPV-7. Surface plots diagrams of three-dimensional data of the indicated cells (arrow) are shown in grey images.
