# Supplementary material for: High eEF1A1 Protein Levels Mark Aggressive Prostate Cancers and the In Vitro Targeting of eEF1A1 Reveals the eEF1A1–actin Complex as a New Potential Target for Therapy
Source: Int J Mol Sci. 2022 Apr 8;23(8):4143. doi: 10.3390/ijms23084143 (PMC9027132; doi:10.3390/ijms23084143)
Supplement: Supplementary file 1 [file ijms-23-04143-s001.zip › Figure S4.pdf]

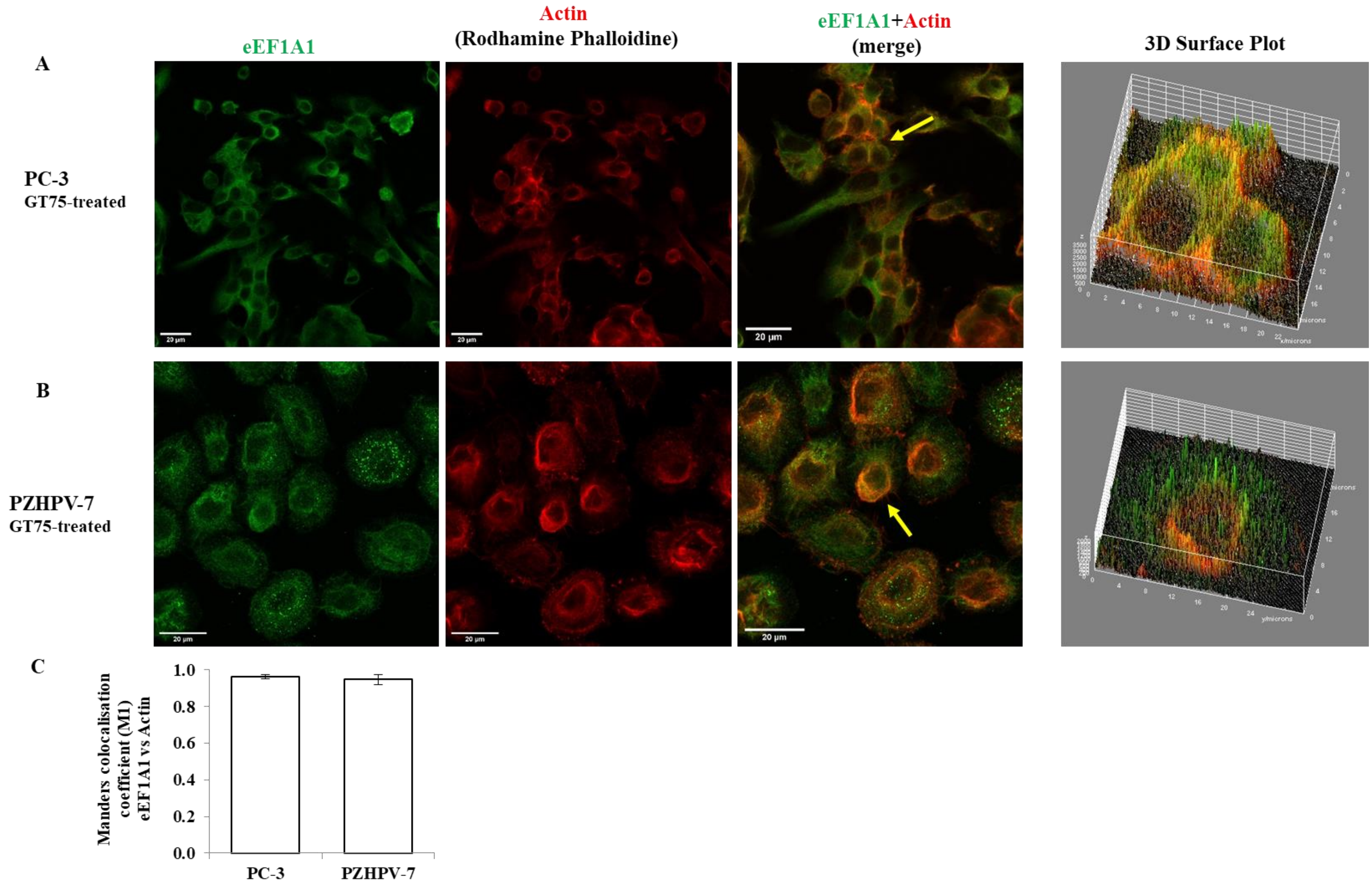

Figure S4: A: Representative immunofluorescence confocal images showing the co-localisation of eEF1A1 (green) with actin (red) in GT75-treated PC-3 3 hours after aptamer (150 nM) transfection; confocal Z-stack images, objective immersion oil, 60x, bar 20µm. B: As in A but with PZHPV-7 treated with GT75 (150nM). C: Spatial proximity estimated by Manders' co-localisation coefficients M1 between eEF1A1 and actin, in PC-3 and PZHPV-7 treated by GT75; data are shown as mean ±SEM; n=11 (PC-3) and n=7 (PZHPV-7). Surface plots are diagrams of three-dimensional data of the indicated cells (arrow) are shown in grey images.
