# Supplementary material for: High eEF1A1 Protein Levels Mark Aggressive Prostate Cancers and the In Vitro Targeting of eEF1A1 Reveals the eEF1A1–actin Complex as a New Potential Target for Therapy
Source: Int J Mol Sci. 2022 Apr 8;23(8):4143. doi: 10.3390/ijms23084143 (PMC9027132; doi:10.3390/ijms23084143)
Supplement: Supplementary file 1 [file ijms-23-04143-s001.zip › Figure S5.pdf]

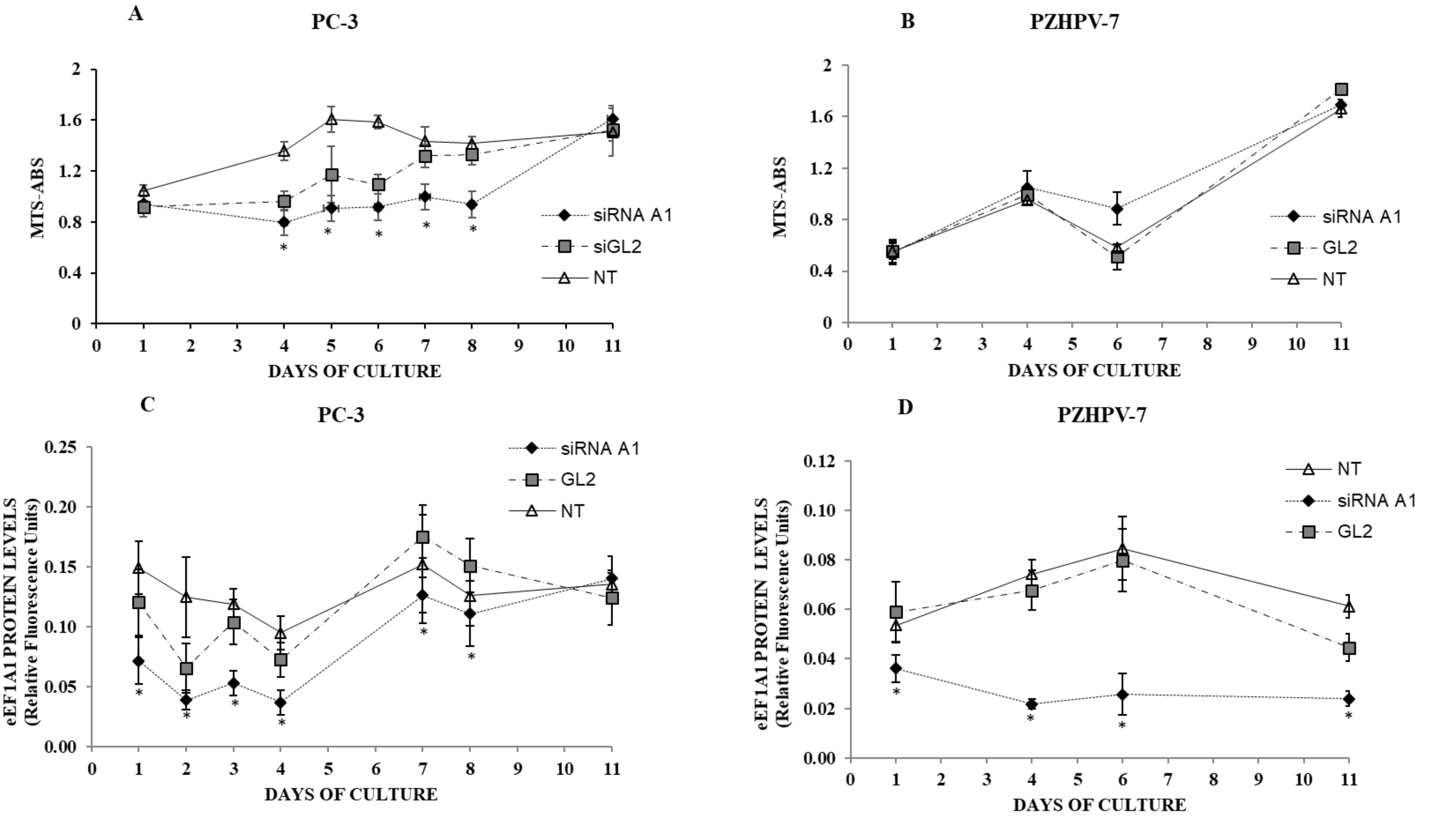

Figure S5: Effects of siRNA-mediated silencing of eEF1A1 on its protein level and cell viability. A-B: effects of siRNA A1 (250 nM) on the viability (MTS assay) of PC-3 and PZHPV-7 different days after siRNA transfection. Data are shown as mean ±SD, n=3. **p*<0.05 compared to control siGL2 treated cells. C-D: reported is the effect on eEF1A1 protein level of the specific siRNA A1 (250 nM) different days after transfection in PC-3 (C) and PZHPV-7 (D), evaluated by in-cell western. NT: non-treated cells, siRNA A1: cells treated with siRNA against eEF1A1 mRNA; siGL2: cells treated with a control siRNA against luciferase mRNA.
