# Supplementary material for: High eEF1A1 Protein Levels Mark Aggressive Prostate Cancers and the In Vitro Targeting of eEF1A1 Reveals the eEF1A1–actin Complex as a New Potential Target for Therapy
Source: Int J Mol Sci. 2022 Apr 8;23(8):4143. doi: 10.3390/ijms23084143 (PMC9027132; doi:10.3390/ijms23084143)
Supplement: Supplementary file 1 [file ijms-23-04143-s001.zip › Figure S6.pdf]

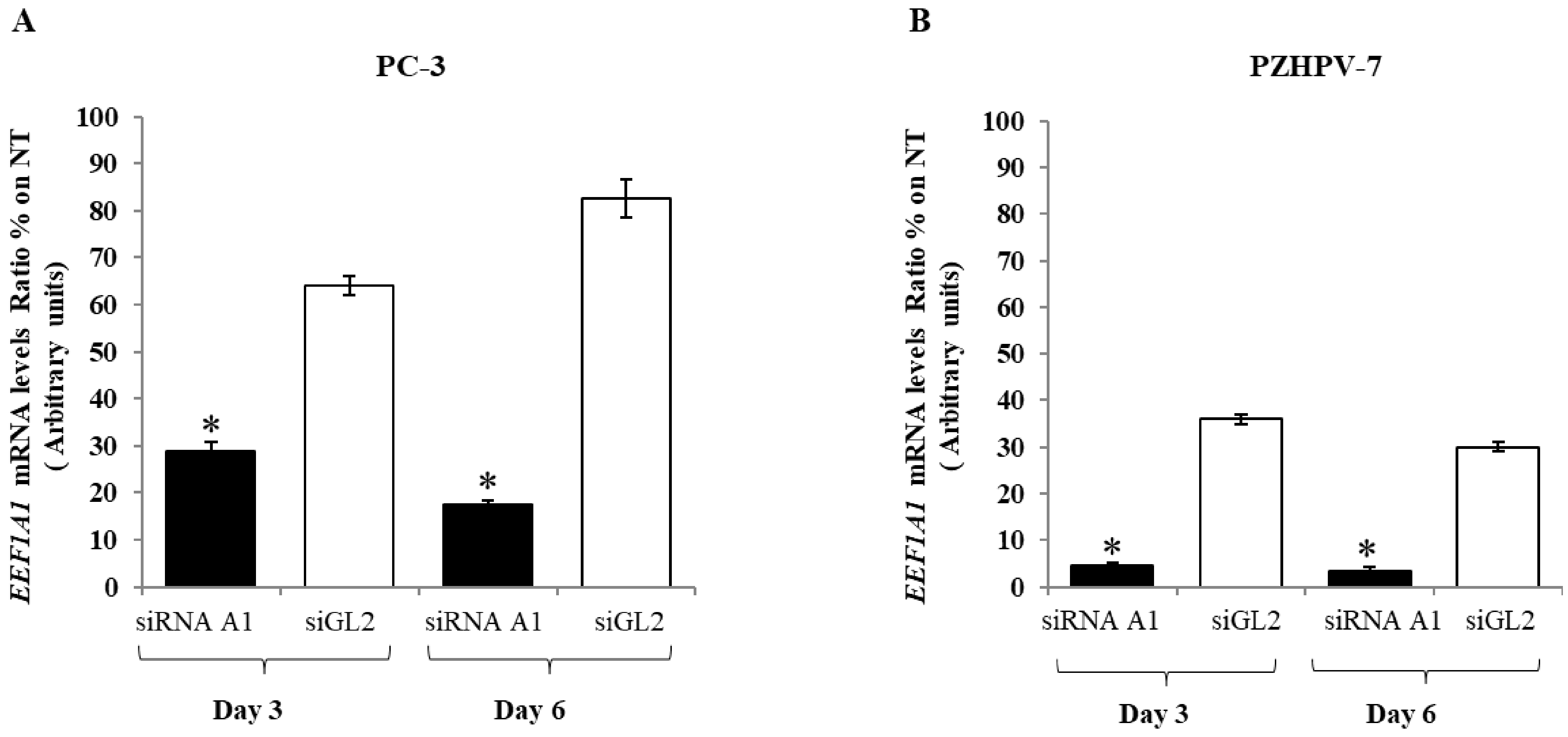

Figure S6: Effect on *EEF1A1* mRNA level of siRNA A1 (targeted against eEF1A1) 3 and 6 days transfection in PC-3 (A) and PZHPV-7 (B) cells. siGL2: control siRNA against luciferase mRNA. Data are expressed as ratio percentage (%) of not treated controls (NT). Data are shown as mean ±SD, n=8*. *p<0.01 compared to GL2 treated cells.
