# Supplementary material for: High eEF1A1 Protein Levels Mark Aggressive Prostate Cancers and the In Vitro Targeting of eEF1A1 Reveals the eEF1A1–actin Complex as a New Potential Target for Therapy
Source: Int J Mol Sci. 2022 Apr 8;23(8):4143. doi: 10.3390/ijms23084143 (PMC9027132; doi:10.3390/ijms23084143)
Supplement: Supplementary file 1 [file ijms-23-04143-s001.zip › Figure S7.pdf]

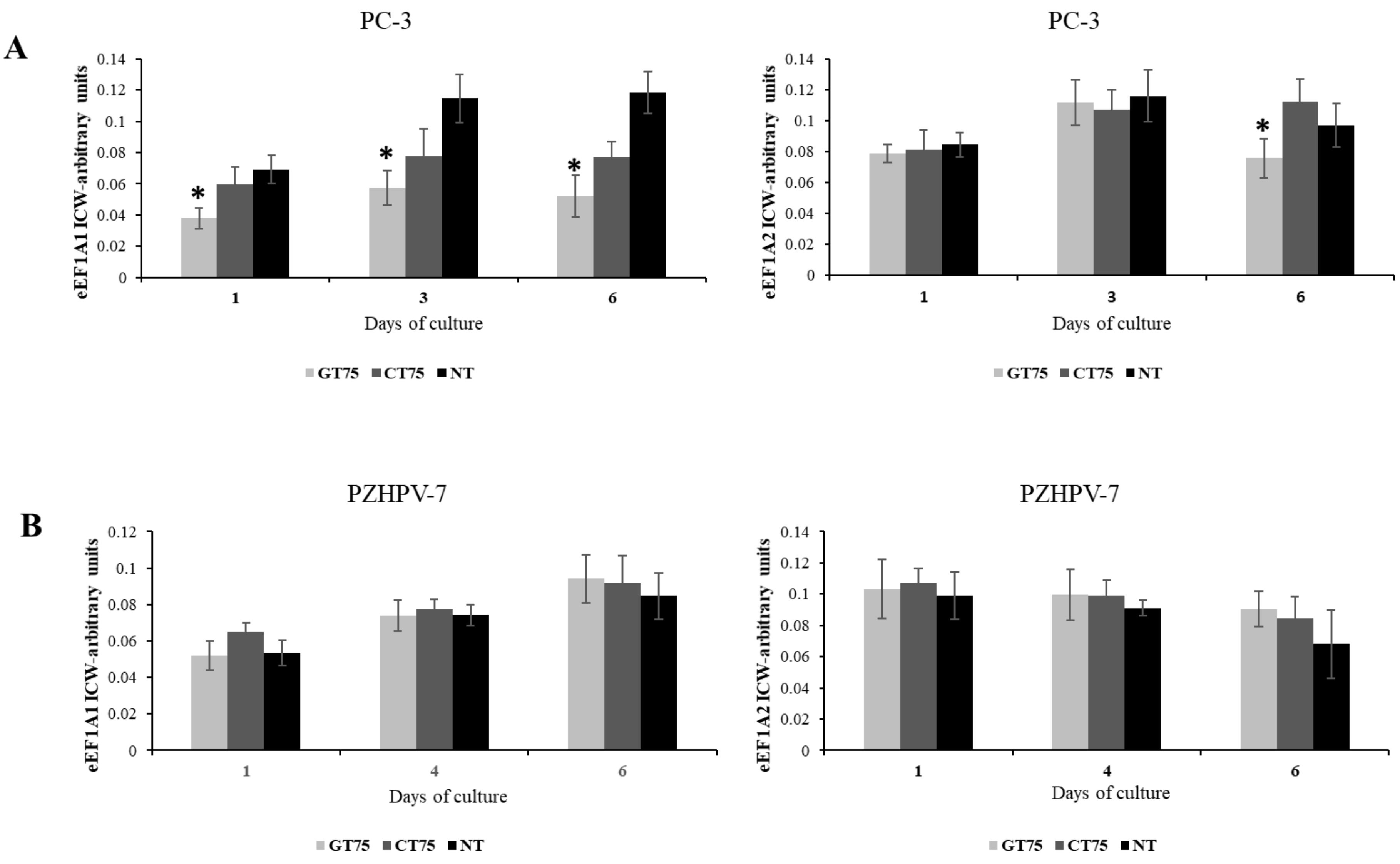

Figure S7: PC-3 cancer cells (A) or non-tumorigenic PZHPV-7 cells (B) were seeded in 96 microtiter well and transfected with 150 nM of GT75 aptamer or of CT75 control. ICW was performed at the indicated days for eEF1A1 and eEF1A2 proteins. NT= non treated cells; *statistically significant at T-test ($0.001<p<0.03$)
