# Supplementary material for: High eEF1A1 Protein Levels Mark Aggressive Prostate Cancers and the In Vitro Targeting of eEF1A1 Reveals the eEF1A1–actin Complex as a New Potential Target for Therapy
Source: Int J Mol Sci. 2022 Apr 8;23(8):4143. doi: 10.3390/ijms23084143 (PMC9027132; doi:10.3390/ijms23084143)
Supplement: Supplementary file 1 [file ijms-23-04143-s001.zip › supplementary_tables.pdf]

**Patients**			**Age at diagnosis**		
Gleason	grade	nr	mean	median	SD
7-8	3+4	10	65.6	66	5.5
	4+3	8	68.4	69	4.1
	4+4	5	70.2	72	3.6
4-6	2+2	9	72	72	3.2
	2+3	2	75	75	10
	3+2	3	69	67	6.2
	3+3	12	69.7	70	7.1
Hyperplasia		23	71.2	72	8

Table S1. Patients'cohort

Variable	Hyperplasia (n=23)	Gleason 4-6 (n=26)	Gleason 7-8 (n=23)	*p*-value
**Age**				
**Median (Min-Max)**	72 (54-88)	71 (56-82)	68 (55-75)	0.11
**eEF1A1 Expression**				Kruskall–Wallis test
**Mean (SD)**	1.9 (1.1)	1.4 (1.1)	2.2 (0.9)	0.044
**Median (Min-Max)**	2 (0-3)	2 (0-3)	2 (0-3)	
**eEF1A1 score (n,%)**				Chi-squared test
**0-1**	8 (34.8%)	12 (46.2%)	3 (13.0%)	0.043
**2-3**	15 (65.2%)	14 (56.8%)	20 (87.0%)	
**eEF1A1 mRNA***	Hyperplasia (n=7)	Gleason 4-6 (n=14)	Gleason 7-8 (n=8)	Mann Whithney test
**Mean (SD)**	10.9 (3.1)	17.0 (7.9)	17.3 (7.1)	0.15
**Median (Min-Max)**	10.8 (6.2-15-7)	16.5 (6.0-31.0)	16.6 (6.2-26.0)	

* *expressed as ratio eEF1A1 mRNA/GAPDH mRNA*

Table S2. Evaluation of eEF1A1 expression in patients'cohort

Variable	Hyperplasia (n=7)	Gleason 4-6 (n=14)	Gleason 7-8 (n=8)	*p*-value
**mRNA**				
**Median (Min-Max)**	10.8 (6.2-15.7)	16.5 (6.0-31.0)	16.6 (6.2-26.0)	0.15

Table S3. Expression of mRNA of eEF1A1in selected samples of the patients'cohort

Recurrence	eEF1A1 categories 0-1	2-3	Total
**0**	13<br>92.86	21<br>70.00	34<br>77.27
**1**	1<br>7.14	9<br>30.00	10<br>22.73
**Total**	14<br>100.00	30<br>100.00	44<br>100.00

Table S4. Relation between recurrence and eEF1A1 score (categorization) in patients'cohort
